# Quantitative Flow Ratio or Angiography for the Assessment of Non-culprit Lesions in Acute Coronary Syndromes: Protocol of the Randomized Trial QUOMODO

**DOI:** 10.3389/fcvm.2022.815434

**Published:** 2022-04-04

**Authors:** Helen Ullrich, Maximilian Olschewski, Khelifa-Anis Belhadj, Thomas Münzel, Tommaso Gori

**Affiliations:** ^1^Department of Cardiology, Cardiology I, University Medical Center Mainz, Mainz, Germany; ^2^German Centre for Cardiovascular Research (DZHK), Standort RheinMain, Mainz, Germany

**Keywords:** acute coronary syndrome, percutaneous coronary interventions, quantitative flow ratio, angiography, fractional flow reserve

## Abstract

**Background:**

Approximately 50% of the patients undergoing percutaneous coronary intervention (PCI) for acute coronary syndromes (ACS) have additional stenotic lesions in non-infarct-related coronary arteries. The decision whether these stenoses require further treatment is routinely based on angiography alone. The quantitative flow ratio (QFR) is a simple non-invasive method that may help quantify the functional significance of these intermediate coronary artery lesions. The aim of our single-center, randomized superiority trial is to test the impact and efficacy of QFR, as compared to angiography, in the treatment of patients with ACS with multivessel coronary artery disease. Primary goal of the study is to investigate 1. The impact of QFR on the proportion of patients receiving PCI vs. conservative therapy and 2. whether QFR improves angina pectoris and overall cardiovascular outcomes.

**Methods and Analysis:**

After treatment of the culprit lesion(s), a total of 200 consecutive ACS patients will be randomized 1:1 to angiography- vs. QFR-guided revascularization of non-culprit stenoses. Patients and clinicians responsible are blinded to the randomization group. The primary functional endpoint is defined as the proportion of patients assigned to medical treatment in the two groups. The primary clinical endpoint is a composite of death, non-fatal myocardial infarction, revascularization and significant angina at 12 months. Secondary endpoints include changes in the SAQ subgroups, and clinical events at 3- and 12-month follow-up.

**Discussion:**

This study is designed to investigate whether QFR-based decision-making is associated with a decrease in angina and an improved prognosis in patients with multivessel disease.

**Trial Registration Number:**

ClinicalTrials.gov Registry (NCT04808310).

## Introduction

Of the patients undergoing PCI for an ACS, ~50% have additional stenotic lesions in non-infarct-related coronary arteries (1). These lesions need to be correctly classified into those hemodynamically non-significant (not needing treatment) and those hemodynamically significant (needing treatment). This decision is commonly performed using angiography with visual assessment of diameter stenosis. Alternatively, a more accurate stratification can be achieved by measuring coronary hemodynamics invasively. A number of studies show that fractional flow reserve (FFR)-guided PCI is superior to angiography guided PCI in terms of mortality, infarction and unplanned revascularizations (2–4). Use of FFR and analogous indices in patients with stable coronary artery disease has been shown to improve outcomes of patients with chronic coronary syndromes (2, 3, 5–7). In the setting of ACS, three randomized trials tested the impact of an FFR-guided approach for the treatment of non–infarct-related coronary artery lesions in ST-segment elevation myocardial infarction (STEMI) patients. While DANAMI-Primulti and COMPARE Acute showed a significant reduction in repeat revascularizations in the FFR group as compared to the angiography group (8, 9), the FLOWER-MI (10) trial did not show a significant advantage with respect to the risk of death, myocardial infarction, or urgent revascularization at 1 year (10). Finally, in the FAMOUS-NSTEMI trial (11), the proportion of patients initially treated with medical therapy was higher in the FFR-guided group compared to angiography-guided group. Importantly, at 12 months, the rate of revascularization was lower in the FFR-guided group (11).

The need of additional time limit the penetration of invasive physiology assessment in clinical routine, particularly in the setting of ACS where patients need to be treated rapidly. Additionally, since they require the use of an intracoronary wire, these methods do not allow *post-hoc*, off-line measurements.

In contrast, QFR is a simple and non-invasive parameter based on three-dimensional quantitative coronary angiography (12) that allows *post-hoc*, off-line analysis without prolonging the intervention in the acute setting. A number of studies have now shown that the results of QFR correlate well with those of FFR, and a recent meta-analysis of nine studies for a total of 1,175 vessels in 1,047 patients reported a pooled sensitivity, specificity, positive and negative likelihood ratio for QFR of 0.89 (95% CI: 0.86–0.92), 0.88 (95% CI: 0.86–0.91), 6.86 (95% CI,: 5.22–9.02), 0.14 (95% CI: 0.10–0.21). The area under the summary receiver operating characteristic (sROC) curve for QFR was 0.94 (13). Further, QFR has been validated by several studies in the context of non-culprit lesions of ACS (14–18).

Recurrence of angina is a common phenomenon, which affects up to 32% of patients in the first year after PCI (19, 20). In the setting of ACS, using data from the MERLIN-TIMI 36 trial, it was shown that 4 months after ACS, ~30% of patients reported monthly angina, more than 15% weekly angina, and more than 4% daily angina (21). Our study aims to investigate whether assessment of non-culprit lesions with QFR improves outcomes of ACS patients with multivessel disease.

## Materials and Methods

### Overview

We investigate whether the use of a computerized functional assessment of stenosis severity based on 3-dimensional reconstruction of coronary anatomy using QFR improves angina pectoris and cardiovascular outcomes in patients with a successfully treated ACS and non-culprit residual intermediate coronary artery stenoses.

### Study Design

The study is a single-center, randomized, parallel, superiority trial to compare two strategies to guide revascularization of non-culprit coronary lesions in ACS patients who have undergone interventional treatment of culprit lesions. The hypothesis of the study is that QFR-guided assessment of non-culprit lesions will be associated with improved discrimination of ischemia-inducing lesions, leading to lower event rates and less angina pectoris at follow-up. The protocol complies with good clinical practice (GCP) and the ethical principles described in the Declaration of Helsinki, has been approved by the local ethics committee and registered under ClinicalTrials.gov (NCT04808310). All patients participating in the study must provide written informed consent. The study, including the initial interventional procedure, all study-related measurements and possible further procedures, will be conducted in the catheterization laboratory of the Department of Cardiology, Cardiology 1 of the University Medical Center Mainz.

### Trial Population

All patients with unstable angina, NSTEMI or STEMI who have received successful PCI (Thrombosis in Myocardial Infarction score of at least 2 and residual stenosis <30%) of all culprit lesions and receive guideline-directed medical therapy will be evaluated. Patients who present at least one non-culprit coronary artery lesion (>30 and <90% by visual estimation in a major epicardial coronary artery or major side branch measuring ≥2.0 mm in diameter) that is judged to be amenable to PCI will be screened for enrolment. Patients with multiple lesions and/or lesions in more than one vessel can be included. Patients will be excluded if any of the following criteria applies: age <18 years; persistent symptoms or evidence of ischemia (troponin raise, ST-changes, angina) following treatment of the ACS-culprit lesion and requiring (re-)intervention of the culprit or other lesions; non-culprit stenoses or patients not amenable to treatment with PCI (e.g., limited life expectancy, small-vessel disease); patients for whom PCI is believed to be an unsafe alternative (e.g., renal failure); any contraindication to PCI according to guidelines; presence of thrombus in the non-culprit lesion; participation in another randomized interventional study interfering with the present protocol; women of child-bearing potential or lactating; previous coronary artery bypass graft (CABG) surgery; recent (within 30 days) unsuccessful PCI; decompensated congestive heart failure (CHF) or hospitalization due to CHF during the last 3 months; left ventricular ejection fraction <30%; severe chronic obstructive pulmonary disease (COPD); severe valvular heart disease; FFR or RFR (resting full cycle ratio) oder iFR (instantaneous wave free ratio) assessment of non-culprit lesions at the time of the index procedure (see Table 1). Patients unable to understand the scope of the study are classified as not able to give informed consent and are ineligible for study enrollment. Likewise, patients unable to consent, e.g., for a neurological damage, are treated as not able to give informed consent and are excluded.

**Table 1:**
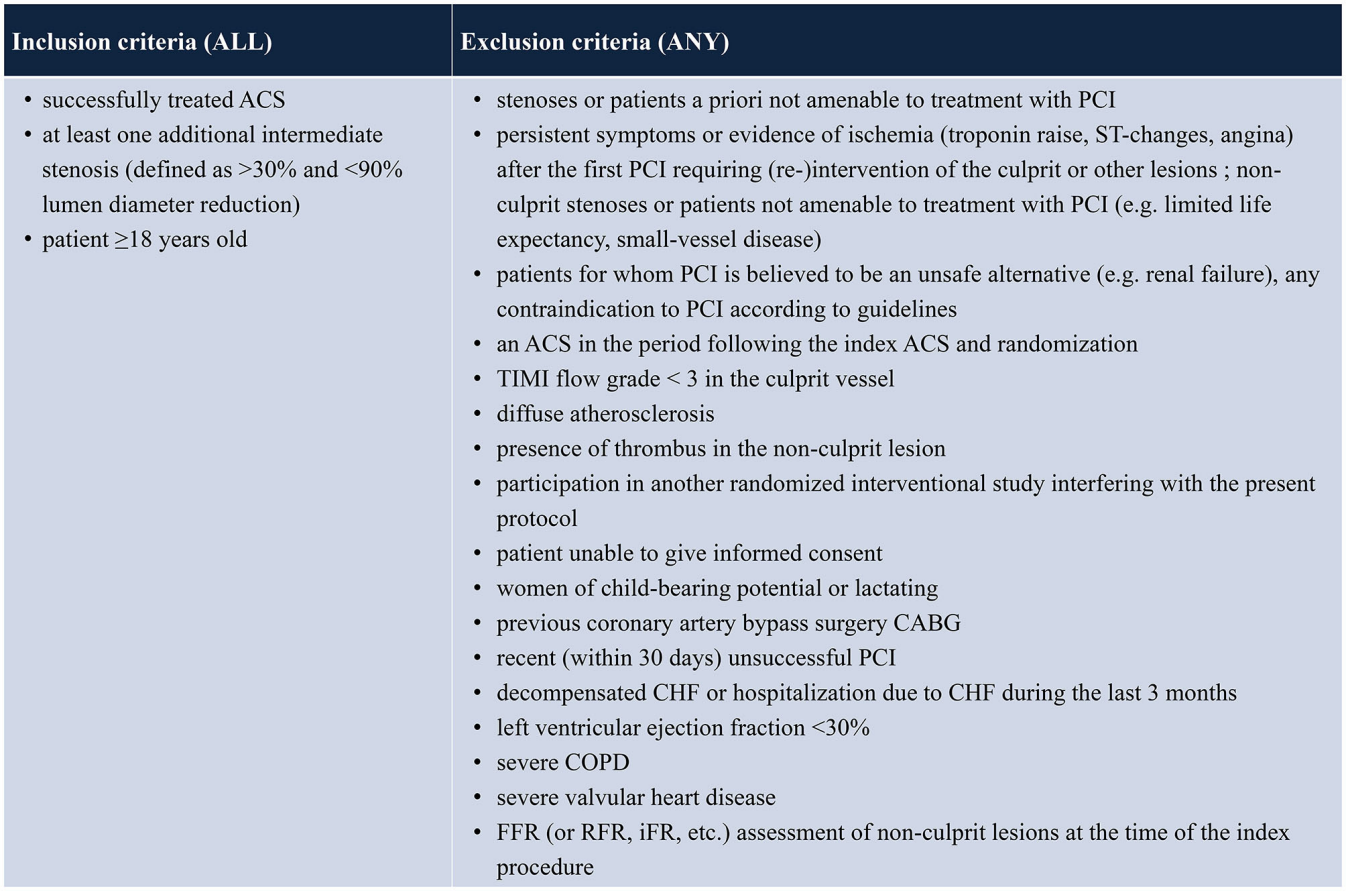
In- and exclusion criteria.

### Study Procedures

#### Consent and Randomization

Patients will be contacted after successful interventional treatment of the ACS, which includes treatment of all culprit lesions (typically >90% diameter stenosis or thrombotic lesions) and guideline-directed medical therapy. If all inclusion and no exclusion criteria are met, consent will be obtained. Prior to randomization, QFR will be measured in all participants in all coronary arteries presenting a lesion with 30–90% diameter stenosis. Patients with vessels with residual QFR <0.80 (residual QFR is the QFR calculated in the absence of the target lesion. A QFR <0.80 assumes that treatment of this/these focal lesion(s) would not remove the source of ischemia, such as in the case of diffuse disease) will be excluded. Thereafter, randomization will be performed in a 1:1 ratio with the use of randomly permuted blocks of 2, 4 or 6. The participants and treating physicians will be blinded to the treatment group allocation. Patients will be randomized to one of two strategies: angiography-guided (reference) or QFR-guided revascularization (experimental). In the reference arm, any decision regarding the treatment of non-culprit lesions will be based on the angiography performed at the time of the index PCI and the physicians will be kept blinded to the outcomes of QFR analysis. This strategy reflects the typical procedures used in daily routine; all lesions believed to be significant at angiography will be treated in one staged session. In patients randomized to QFR-guided revascularization, all lesions (one or more per vessel) associated with a vessel QFR <0.80 and a residual QFR >0.80 will be treated in one staged session (13). The treatment plan will be scheduled (according to angiography or QFR) by study investigators not involved in patient care. Only experienced medical staff, certified to use the software with the QFR system, will perform the measurements. Analyses will be conducted with QAngio XA 3D from Medis Medical Imaging Systems BV (Leiden, the Netherlands).

#### Staged Revascularization

Staged PCI will be performed 4 weeks after the first PCI performed in the setting of the ACS. PCI will be performed with newer-generation drug eluting stents at the operator's discretion. Use of intracoronary imaging is allowed, but not for the assessment of the relevance of the stenosis (i.e., not for overruling the decision made by angiography or QFR). FFR or other invasive hemodynamic assessments prior to PCI are not allowed.

Reference vessel diameters calculated by the QFR software will be used.

#### SAQ Questionnaires

The first SAQ questionnaire will be administered before the staged revascularization, i.e., ~4 weeks after the ACS event. The SAQ-7 provides a quantitative analysis of 5 domains (physical limitation, angina stability, frequency, treatment satisfaction, and quality of life) reflecting the impact of angina on patients' health status during the previous 4 weeks. Scores for each domain go from 0 to 100, with 100 defining absence of limitations. The SAQ score at 1 month after ACS correlates with the subsequent incidence of mortality, hospitalization, and resource use (22). The SAQ-7, along with clinical follow-up data (death, re-hospitalization, re-intervention) will be collected at 3 and 12 months after protocol-mandated complete revascularization during office or telephone visits.

### Study Endpoints

*Primary endpoint (functional): the primary endpoint (functional) is* the proportion of patients assigned to medical treatment in the two groups (QFR vs. Reference) (see Table 2).

**Table 2:**
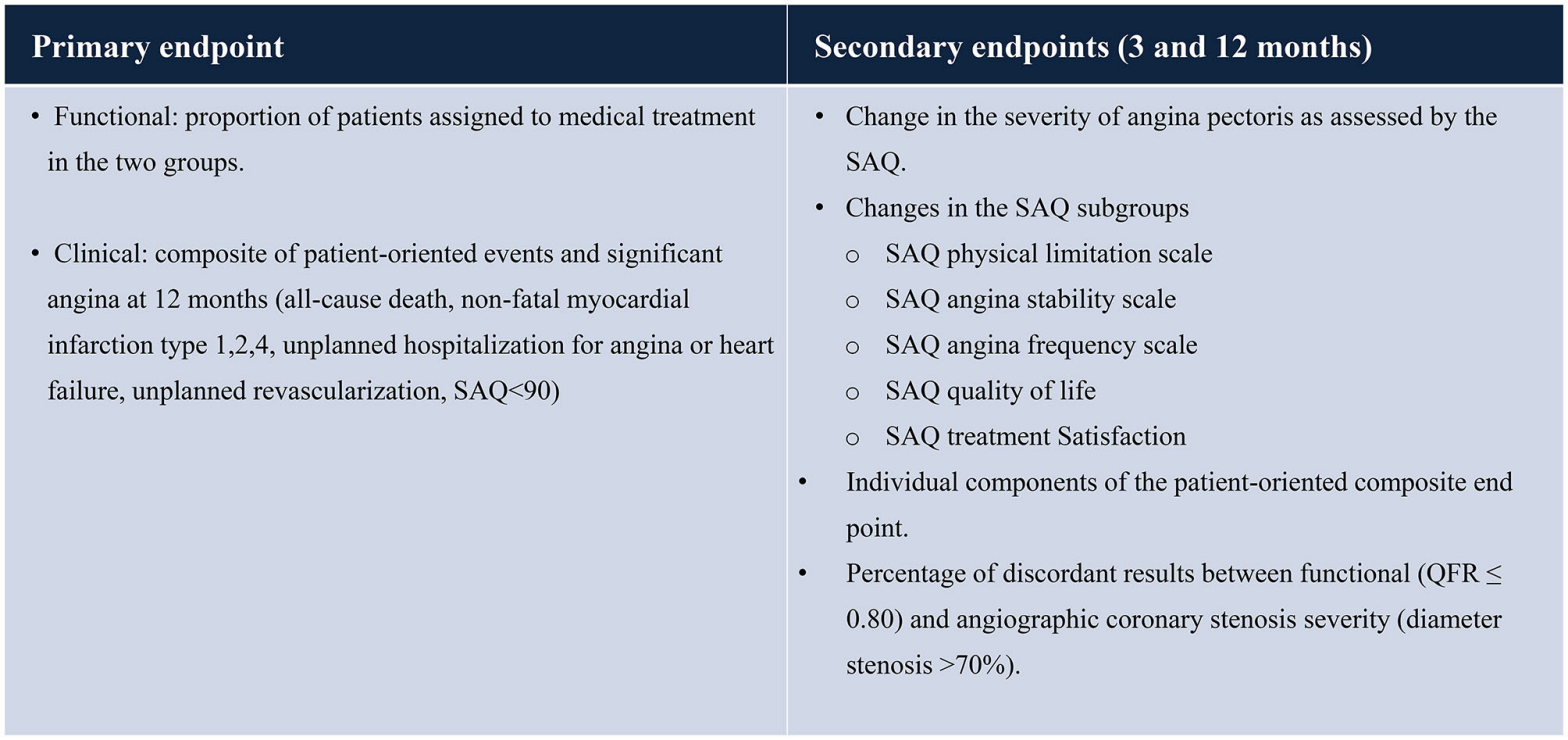
Study endpoints.

*Primary endpoint (clinical):* the primary endpoint (clinical) is a composite of patient-oriented events and significant angina (all-cause death, non-fatal myocardial infarction including type 1, 2, 4, unplanned hospitalization for angina or heart failure, unplanned revascularization, SAQ <90) at 12 months.

*Secondary endpoints:* the secondary endpoints include the following (at 3 and 12 months):

– the percentage of discordant results between functional (QFR ≤ 0.80) and angiographic coronary stenosis severity (diameter stenosis >70%).
– the change in severity of angina pectoris as assessed by the SAQ summary score.
– changes in the SAQ-7 domains (SAQ Physical limitation scale; SAQ angina stability scale; SAQ angina frequency scale; SAQ quality of life; SAQ Treatment Satisfaction).
– incidence of a patient-oriented composite endpoint and its components (cardiovascular death, non-fatal myocardial infarction including peri-procedural, unplanned revascularization, unplanned hospitalization for angina, SAQ summary score <90) and its individual components.

### Sensitivity Analysis

A sensitivity analysis will test the primary and all secondary endpoints in patients with intermediate stenoses >50% (i.e., patients with intermediate non-culprit lesions 30–50% will be excluded from this analysis).

### Adjudication of Events

All clinical events will be adjudicated by a clinical events committee composed by members who are blinded to the allocation group.

### Recruitment

Patient recruitment is performed within the patients treated at the Department of Cardiology, Cardiology 1 of the University Medical Center Mainz.

### Statistics

Statistical analysis will be performed with the MedCalc Software (Ostend, Belgium) and SPSS version 24 (SPSS, Chicago, Illinois).

All subjects who signed informed consent and are assigned a randomization number are considered as ITT subjects, even if they have not received trial treatment. All randomized subjects who were randomized and have a 12-month assessment of the primary endpoint variable will be included in the modified Intention-to-treat (mITT) population. This population is the primary analysis population. Within the mITT population subjects will be assigned to the treatment to which they were randomized. Missing values of SAQ-7 will not be replaced or imputed for the primary analysis.

### Power Calculation

Functional endpoint: For the sample size calculation, the results of the FLOWER-MI trial will be used. In this study, which enrolled multivessel disease patients with at least one non-culprit lesion >50%, PCI was performed in 388 of 586 patients (66.2%) in the FFR-guided group and in 560 of 577 patients (97.1%) in the angiography-guided group; 366 of 826 (44.3%) lesions in the FFR group were reclassified from angiographically significant to non-significant based on FFR. Of note, although we allow patients with lesions >30% in our study, we foresee that [based on the results of the FLOWER-MI (10)], about 97% of the participants in our study will have at least one lesion >50%. Conservatively, we hypothesize a rate of decision to not perform PCI in our angiography group 4 times higher than that observed in the FLOWER-MI study (i.e., 13%). In the QFR group, we hypothesize a conservative decision in 33% (similar to that observed in the FLOWER-MI study). Based on this hypothesis, 176 patients are required to have a 90% chance of detecting, as significant at the 5% level, a decrease in the primary outcome measure from 33% in the QFR group to 13% in the angiography group.

Clinical endpoint: The clinical endpoint combines hard endpoints and angina: in the FORZA trial, comparing a FFR-guided with a OCT-guided PCI strategy, a composite of MACE + significant angina (all-cause death, non-fatal myocardial infarction, target vessel revascularization, SAQ <90) was taken. The incidence of this endpoint was 14.8% at 1 year in the FFR group (23). The incidence of the same endpoint in the FAME trial was 32.4% at 1 year in the angiography group (3). Given these assumptions, 176 patients are required for a power of 80% and an alpha error of 5% to observe a difference assuming an incidence of 14.8% in the QFR group and 32.4% in the angiography group.

With regards to the change in SAQ, this endpoint was also used in a number of studies, including the ISCHEMIA trial (24): In the TARGET FFR study (25), 260 patients received first angiographically guided PCI and were then randomized (1:1) to a physiology-guided incremental optimization strategy or a blinded coronary physiology assessment. SAQ was a secondary endpoint in this study. A direct comparison with our protocol is impossible (all patients received PCI in TARGET FFR, while our hypothesis is that QFR will prevent futile PCIs and increase necessary ones), however the change in SAQ summary score at three months in both groups was in the range of 21 ± 25. Patients with a larger FFR change had a higher 3-months SAQ (86 ± 24 vs. 72 ± 30, *P* = 0.02 with *n* = 47 and 52 per group). If a similar difference were to be observed in our study, 144 patients would be required with a 90% 1-beta and a 5% alpha. In the FORZA study (23), the SAQ similarly improved from ~82 to ~98 (data reported only in online Figure 1). If we assume a SAQ of 98 in the QFR group and 86 in the reference group, with a SD of 21, 130 patients would be necessary.

**Figure 1:**
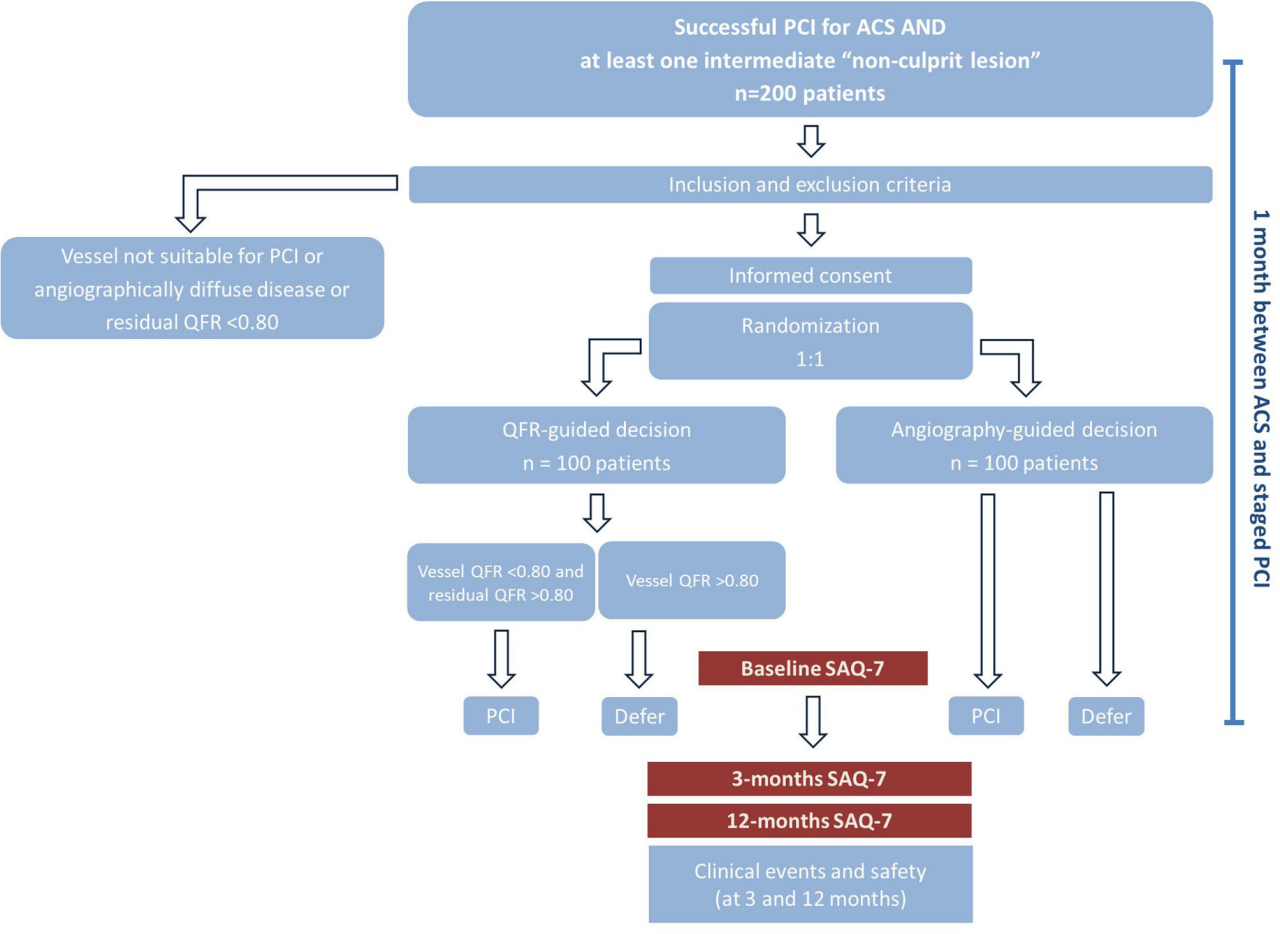
Study procedures.

In another paper of the Glasgow group (26), the authors enrolled 104 patients with angina with 1:1 randomization to PCI or OMT. The primary outcome was angina status at 3 months using the Seattle Angina Questionnaire (SAQ). After 3 months of follow-up, compared with patients treated with OMT only, patients treated by PCI and OMT had greater improvements in SAQ angina frequency (21 ± 28 vs. 10 ± 23; *p* = 0.026). So also in this case the observed improvements in SAQ appear to be larger than those conservatively predicted in our protocol.

Given the above data, we can safely conclude that our sample size of ~200 will allow testing the hypothesis of a difference in SAQ between groups.

### Hypothesis

A = Change in the SAQSS after 3 months to baseline with QFR-guided treatment

B = Change in the SAQSS after 3 months to baseline with reference treatment

Hypotheses: H0: A = B; H1: A ≠ B (Overall type I error rate = 5% (two-sided) with a power = 90%).

Based on these assumptions, the sample size is 178. We plan to recruit 100 patients per group.

### Statistical Analysis of Primary and Secondary Endpoints

Data will be presented as counts (percentages) and mean ± SEM as appropriate and will be analyzed using parametric or non-parametric tests. The primary functional endpoint will be assessed as difference in proportions and the relative risk between groups will be estimated with exact 95% confidence intervals and *P*-values. The proportion of patients with primary functional clinical endpoint and other binary outcomes will be analyzed using the same methods, and time to events within 12 months will be compared between groups using log-rank tests. A multivariable stepwise linear regression (*p* < 0.2 to enter, *p* < 0.1 to stay) analysis will be used. Covariates will include age, sex, diabetes, smoking, renal function, previous myocardial infarction, type of acute coronary syndrome presentation, body mass index, vessel(s) involved. The grouping factor (QFR vs. angiography) will be forced into the model. A *p*-value <0.05 (2-sided) will be considered statistically significant. All secondary endpoints will be analyzed by descriptive statistics and appropriate exploratory *p*-values.

### Safety Analyses

The procedures related to this study do not modify the risks linked with any intracoronary procedure and a difference between the two groups is not expected. The following clinical events will be recorded, as they represent the standard clinical endpoints that are assessed to determine the safety and outcome after interventional procedures. All clinical data will be collected at discharge from hospital and during follow-up contacts at 3 and 12 months and will be evaluated by a data monitoring and safety board composed by physicians who are otherwise not involved in the study.

Periprocedural cardiac biomarker release is defined as (27):

1. In patients undergoing PCI with normal (≤ 99^th^ percentile URL) baseline cardiac troponin (cTn) concentrations, elevations of cTn >5 times the 99th percentile URL occurring within 48 h of the procedure.
2. In patients undergoing PCI with elevated baseline, where two measures are available showing stable or falling values, a rise of >20% after PCI.
3. In patients with elevated cTn levels before PCI and raising cTn or only one cTn value available, this endpoint cannot be evaluated.

Target lesion failure: defined as a composite of cardiovascular death, target-vessel myocardial infarction.

Target lesion revascularization: any (including attempted) repeat revascularization with either balloon angioplasty, stenting, or coronary artery bypass grafting, within the previous treated vessel segment including the 5 mm proximal or distal. Where there is doubt about the need for re-intervention, physicians are strongly recommended to use FFR of invasive imaging to ascertain whether re-intervention is required.

Death: Cause of death will be considered cardiac unless specified otherwise. In the primary analysis all deaths will be compared. The Coordinating site must be notified of a patient's death within 3 days of its knowledge. Data relating to the patient's death should be recorded. A copy of the death certificate with anonymized study identification number but with the patient's name removed should be sent to the coordinating site within 3 weeks of the patient's death. Post mortem results if available should follow as soon as possible.

Cardiac death: Any death due to proximate cardiac cause (e.g., MI, low-output failure, fatal arrhythmia), un-witnessed death and death of unknown cause, and all procedure-related deaths, including those related to concomitant treatment.

Vascular death: Death caused by non-coronary vascular causes, such as cerebrovascular disease, pulmonary embolism, ruptured aortic aneurysm, dissecting aneurysm, or other vascular diseases.

Non cardiovascular death: Any death not covered by the above definitions, such as death caused by infection, malignancy, sepsis, pulmonary causes, accident, suicide, or trauma.

Clinically relevant myocardial infarction: The Universal definition of Myocardial Infarction (Revision 2018) will be used to define clinically relevant myocardial infarction (MI) in this study (28).

### Confidentiality

Patient data will be pseudonymized and collected by the study team. Pseudonymized patient data will be stored digitally and only accessible to the members of the study team. After 10 years of storage, the data will be destroyed. It is not intended to give study participants' data to a third party. All data will be analyzed after the last patient is discharged from index hospitalization. No interim analysis is intended except for the safety evaluation performed by the Data Safety Monitoring Board consisting of two physicians not affiliated with the study. In case a study participant withdraws consent after having his data collected from him, the patient's data will be anonymized.

### Monitoring

External Monitoring is not planned.

### Ethics and Publication Policy

The protocol has been approved by the local state medical association's ethics committee. The procedures outlined in this protocol for the conduct, evaluation and documentation of this study are intended to ensure that all persons involved in the study comply with GCP and the ethical principles described in the Declaration of Helsinki. The study will be conducted in accordance with local legal and regulatory requirements. The requirements of the German Medicines Law, the GCP regulation and the Federal Data Protection Law are adhered to.

The PI of this study is committed to the unrestricted and widespread dissemination of all primary and secondary endpoint results and tertiary analyses. At the conclusion of the study, an abstract reporting the primary results will be prepared by the investigators and presented. A publication will similarly be prepared for publication in a reputable scientific journal. No outcome data from either endpoint will be published or made available to the Investigators in any form, unless decided otherwise from the data safety and monitoring board, until the discharge of the last patient enrolled into the study. Following analysis and presentation of the primary endpoint results, active participation of all study group members will be solicited for data analysis and abstract and manuscript preparation and therefore included as co-authors. Submission of all abstracts and publications regarding the primary endpoint and secondary endpoints from the study requires approval by the PI after review by all members of the study group.

### Trial Status

Data collection is ongoing. The first patient was enrolled in October 2020. We expect the study to be completed in May 2022.

### Financing

The study will be financed by own means of the Department of Cardiology, Cardiology 1, University Medical Center Mainz (=Sponsor) and means of the W3-Professorship of Translational myocardial and cardiovascular function. No third-party funds are planned.

## Discussion

Multivessel disease occurs in 40–65% of patients with acute myocardial infarction. As a consequence, angina after successful PCI of culprit lesions is frequent (29) and several studies showed that incomplete revascularization may be associated with an increased risk of adverse clinical outcomes (1, 30, 31).

Therefore, it is particularly important to evaluate the hemodynamic significance of the remaining non-culprit lesions, because patients do not benefit from treatment of hemodynamically non-significant stenoses in terms of prognosis or symptoms (12, 32). Large studies demonstrated that the use of FFR is superior to angiography alone in this regard and that FFR-guided PCI improves clinical outcomes (2, 12, 33), while others have not (34, 35). Independently of its prognostic impact, a limitation to the use of wire-based FFR in patients with acute coronary syndrome is that measurement requires additional time in an acute setting and a *post-hoc* offline analysis is not possible. Addressing this problem, our study investigates whether QFR guidance provides a better estimate of the severity of non-culprit lesions compared with angiography. Of importance, Tebaldi et al. recently reported that QFR, Pd/Pa and DFR are equivalent to the gold standard FFR in the discrimination of non-culprit lesions requiring revascularization in patients with NSTEMI who have received PCI of all culprit lesions (18). Our study tests the hypothesis that QFR could provide a better stratification of patients/lesions requiring PCI for non-culprit lesions as compared to angiography, leading to more accurate identification of lesions requiring PCI and to reduced angina during follow-up.

QFR can easily be measured without the need for pressure wires and hyperemic agents. Therefore, QFR could be a useful, timesaving, and non-invasive method to assess the physiological severity of intermediate coronary stenosis (36). In a previous study from our group, we demonstrated that QFR identifies hemodynamically relevant stenoses in about 10% of the patients who have undergone diagnostic coronary angiography and have been discharged with a diagnosis of intact coronaries. In the same study, we demonstrated that a QFR ≤ 0.80 was the strongest predictor of events (HR 3.14, 95%CI 1.78–5.54, *p* = 0.0001), an association which was maintained in several sensitivity analyses (37).

Our study has several limitations. First, the study population is relatively small and the study is not designed to test hard clinical endpoints. Second, the presence of diffuse spasm at the time of an ACS might lead to an overestimation of non-culprit lesions in both groups. In a recent study by Sejr-Hansen et al., QFR performed in the setting of a ACS showed however a very good correlation with staged QFR and also a good diagnostic performance as compared to staged FFR (38). Based on these data, QFR performed in the acute setting appears to maintain its clinical validity. Third, the primary endpoint is assessed at 12 months after protocol-mandated complete revascularization; complaints due to myocardial ischemia, which may occur after a period of more than 1 year, will not be recorded. However, data from the ischemia trial appear to suggest that the incidence of angina remains constant during longer follow-up periods (39).

## Data Availability Statement

The original contributions presented in the study are included in the article/supplementary material, further inquiries can be directed to the corresponding author/s.

## Ethics Statement

The studies involving human participants were reviewed and approved by Ethik-Kommission der Landesärztekammer Rheinland-Pfalz. The patients/participants provided their written informed consent to participate in this study.

## Author Contributions

TG, MO, and HU contributed intellectually to the design and planning of the study. The initial study protocol was written by TG and MO and revised during the course by TG, MO, and HU. TG, MO, HU and K-AB contributed to the collection and interpretation of data. The manuscript was written and corrected by TG and HU. All authors critically reviewed the final draft of the manuscript for intellectual content and approved the final manuscript.

## Conflict of Interest

TG has received speaker fees and grant support from Abbott Vascular, Neovasc, Boston Scientific, Bayer, Astra Zeneca, SMT (not in relationship with this research). He is principal investigator of the DZHK, funded by the Ministry of Research, Germany. The remaining authors declare that the research was conducted in the absence of any commercial or financial relationships that could be construed as a potential conflict of interest.

## Publisher's Note

All claims expressed in this article are solely those of the authors and do not necessarily represent those of their affiliated organizations, or those of the publisher, the editors and the reviewers. Any product that may be evaluated in this article, or claim that may be made by its manufacturer, is not guaranteed or endorsed by the publisher.

## Abbreviations

ACS: Acute Coronary Syndrome
aPTT: Activated Partial Thromboplastin Time
CABG: Coronary Artery Bypass Graft
CHF: Congestive Heart Failure
COPD: Chronic Obstructive Pulmonary Disease
cTn: Cardiac Troponin
ECG: Electrocardiogram
FFR: Fractional Flow Reserve
GCP: Good Clinical Practice
iFR: Instantaneous Wave Free Ratio
INR: International Normalized Ratio
ITT: Intention-To-Treat
MI: Myocardial Infarction
NSTEMI: Non-ST-Segment Elevation Myocardial Infarction
PCI: Percutaneous Coronary Intervention
QFR: Quantitative Flow Ratio
RFR: Resting Full Cycle Ratio
SAQ: Seattle Angina Questionnaire
SAQSS: Seattle Angina Questionnaire Summary Score
SEM: Standard Error Of The Mean
STEMI: ST-Segment Elevation Myocardial Infarction
TIMI: Thrombolysis in Myocardial Infarction (Study Group).
